# Prevalence and risk factors of obstetric fistula: implementation of a need-based preventive action plan in a South-eastern rural community of India

**DOI:** 10.1186/s12905-020-00906-w

**Published:** 2020-03-04

**Authors:** Dharitri Swain, Swayam Pragyan Parida, Saubhagya Kumar Jena, Mahasweta Das, Hrushikesh Das

**Affiliations:** 1 College of Nursing, AIIMS Bhubaneswar, Bhubaneswar, Odisha India; 2 Department of Community Medicine & Family Medicine, AIIMS Bhubaneswar, Bhubaneswar, Odisha India; 3 Department of Obstetrics & Gynaecology, AIIMS Bhubaneswar, Bhubaneswar, Odisha India; 4 College of Nursing, ICMR Project, AIIMS Bhubaneswar, Bhubaneswar, Odisha India; 5 College of Nursing, DST Project, AIIMS Bhubaneswar, Bhubaneswar, Odisha India

**Keywords:** Vesico-vaginal fistula, Recto-vaginal fistula, Urine incontinence, Obstetric morbidities

## Abstract

**Background:**

The study was conducted to estimate the prevalence and risk factors of obstetric fistula in the rural area of the south eastern community of India and the training of community health workers for its prevention.

**Methods:**

A population-based cross-sectional analytical study was conducted in the south eastern rural community of India. A total of 3939 women were included in the study and Probability proportional to size sampling was used in the study. Frequency distribution and logistic regression were computed to analyse the data using STATA version 11.2.

**Results:**

Out of 3939 participants interviewed, 23.7% women reported obstetric fistula symptoms whereas after clinical diagnosis and speculum examination the obstetric morbidity pattern was: Obstetric fistula 0.3%, stress urinary incontinence 20.0%, pelvic inflammatory diseases 1.2%, uterine prolapse 1.4% and urinary tract infection 3.8%. The awareness level of the rural women regarding the obstetric fistula was assessed by a structured knowledge questionnaire and found to be very poor, hence community based fistula training was implemented among community health workers as a health system based strategy for its prevention. Obstetric fistula found to be more prevalent among women of poor educational level, low socioeconomic status, less no of antenatal visits, delay in accessing the emergency obstetric care and prolonged labour (*p* ≤ 0.05).

**Conclusion:**

Finding of the study indicated that the prevalence and risk of developing obstetric fistula was associated with less number of antenatal visits, prolonged labour, delay in timely intervention, delay in accessing emergency obstetric care and more number of movements from home to the delivery place. Finally, our study suggests that emphasis needs to be placed on training of community health workers to facilitate early screening for identification and referral of women with obstetric fistula**.**

## Background

On a global scale, obstetric fistula (OF) remains a significant obstetrical problem in low-resource countries and found to be one of the most visible indicators of maternal morbidity. Obstetric fistula is closely related to prolonged and obstructed labor where emergency obstetrical care is inaccessible and unavailable [1]. Obstructed labor injury complex can lead to a host of physical and psychological injuries and who survive develop a life-altering birth injury i.e. obstetric fistula. A woman with obstructed labor develops a fistula when the presenting fetal part compresses continually the birth canal tissue, bladder base, urethra or sometimes rectum causing ischemia and necrosis of the tissue. Countries with high incidence of maternal mortality also have high rates of obstetric fistula for similar reasons [2]. The obstetric fistula is still largely neglected in the low resource countries and developing world, especially in India where women’s health get compromised due to poor socio-economic status of the community [3]. It is more prevalent among some of the most marginalized members of the population; poverty, teenage pregnancy, early marriage, poor socio-economic status, uneducated girls and women in remote regions of the world, and hence it has remained a ‘hidden’ condition [4, 5].

Reliable data on the prevalence and burden of obstetric fistula is scarce. The commonly quoted prevalence estimate is two million cases worldwide and 50,000–100,000 new cases each year. However, most studies are facility-based and the few population-based and may not have not been suitable to accurately identify obstetric fistula [6].

Apart from a few hospital-based studies, there are less epidemiological data on maternal morbidity on fistula in India. UNFPA 2003 report indicated an overall fistula prevalence of 2.2% (range 0.3–7.6%) in India based on four community surveys carried out during 1989–1993 [7, 8]. There are limited studies on the prevalence and incidence of obstetric fistula and obstetric fistula related other information in India. Reliable data on obstetric fistulas are scarce in the south-eastern rural community of India, Odisha; with a gap in knowledge about the extent of the problem. However, the country’s high maternal mortality ratio and its high adolescent fertility rate (almost a third of births occur to girls aged 15–19 years) reported in Odisha, it is to be expected that obstetric fistula among reproductive age group is a significant problem in this locality, where women are highly dependent on male and difficult for them generally to access health care even in emergency situations. There socio-cultural aspects of fistula and its consequences to those having to live with the condition are not explored and reported well. A population based strategy must be introduced in order to assist the public to appreciate and understand the consequences of living with fistula, particularly its psychosocial consequences [7].

This study was an effort towards identifying the geographical areas of high prevalence of obstetric fistula in the south-eastern rural community of India and to propose effective community health interventions that can help to prevent the condition with a long-term goal of eradicating the condition. A better understanding of the prevalence of obstetric fistula is crucial for targeting of services in striving to achieve a ‘fistula-free generation’ by eradicating obstetric fistula and minimizing all other causes [9]. Therefore, the objectives of the study were 1) to study the prevalence rate of Obstetric Fistula in the south-eastern rural community of India, 2) to determine associated factors of Obstetric Fistula among reproductive age group population, 3) Public health intervention to prevent obstetric fistula among the poor community.

## Methods

### Study setting

The study was conducted at Khurdha district, Odisha which is the eastern rural community of India. The district has a total rural population of 11,67,357 of which 48% are female. The district has 1534 villages which covers 2,47,304 rural households [10]. It has been a long time since intervention of improving maternal health was implemented in this rural district but there was a paucity of evidence on women suffering from labour related complication like obstetric fistula Hence, the study area was selected to fill this gap and gather the information about prevalence of OF among rural women and its associated factors.

### Study design and duration

A population-based cross-sectional analytical study was conducted in the eastern rural community of India to estimate the prevalence, risk factors, health-seeking behaviour and knowledge on obstetric fistula in the south-eastern rural community of India. Along with this a wide range training program for community health workers was also been conducted regarding prevention, diagnosis, treatment and rehabilitation of obstetric fistula. The study was carried out for 26 months period (1st January 2017 to 28th February 2019) after taking authorized ethical permission of community stakeholders.

### Sampling criteria

The study population was women, who are married and under the reproductive age group (15–49) with proven fertility (had at least one abortion or live birth or stillbirth) were included in the study. Women who refused to give written ethical consent were excluded.

### Sample size calculation

The sample size was calculated based on the standard prevalence sample size formula at the 95% confidence level with an estimated proportion of 2% and the desired precision of 0.5% to select 3939 participants.

### Sampling techniques

Due to differences in the proportion of the population in the various committees, a probability proportional to size (PPS) cluster sampling method was applied to achieve a representative sample. Purposively one rural block was selected in the south-eastern rural community of India; these were again divided into smaller population units called the village or cluster. The sample was selected with a two-stage sampling; in the first stage, 132 clusters were selected randomly from a sampling frame of all villages based on the average sample size in each cluster [11]. In the second stage, several households were selected randomly in each cluster based on the sample size. Using this method, the first sampling unit was the cluster and second sampling unit was households that were randomly selected, in each household, one eligible person was interviewed.

### Data collection instruments and method

The study tool was a pre-screening interview schedule of obstetric fistula comprised of all closed-ended questionnaires which inquired participant’s basic demographic characteristics along with self-health status report, reproductive health history, reproductive knowledge, perception and health-seeking practice **(**Supplementary file 1**)**. Fistula symptoms were identified by self reported pre-screening questionnaire by conducting field survey [12, 13]. Fistula camps were conducted frequently in the respective community health centres for final screening of cases those are identified in field survey followed by a vaginal speculum examination to confirm that the vaginal loss is urine draining through the anterior vaginal wall [14]. Clinical criteria for obstetric morbidity were used for finding the morbidity pattern of cases **(**Table 1**)**.

**Table 1:** Clinical criteria for chronic obstetric morbidity [2, 15]

Sl.No	Morbidities	Symptoms	Signs
1.	*True Incontinence* Obstetric Fistula • Vesico- vaginal Fistula • Rectro- vaginal Fistula	• Continuous leaking of urine (incontinence) • Foul/offensive odor of urine • Incontinence of liquid stool/flatus	• Vulval skin (redness, ulceration) • Palpation- Size,number,scarring • *Speculum examination* Fistula can be visualized • *Speculum examination* Fistula- exposing posterior vaginal wall • Rectal examination
2.	*Functional Incontinence* Stress urinary incontinence (SUI).	• Involuntary loss of urine • Sneezing • Coughing	• Involuntary leakage from the urethra • Leakage showing with abdominal pressure, usually a sensation of urge • Incontinence due to contraction of the detrusor.
3.	Pelvic inflammatory diseases	• Lower abdominal pain • Increased vaginal discharge • Menorrhagia • Low backache • Dysmenorrhoea	*Per vaginal examination* • Presence of abdominal/uterine tenderness • Cervical motion tenderness • Adnexal tenderness
4.	Uterine prolapse	• Dragging discomfort • Something coming out from the vagina • Low backache • Vaginal discomfort • Vaginal discharge	*Speculum Examination* Patients asked to cough/bear down, decent of an anterior vaginal wall below its natural position, Decent of uterus below its natural position.
5.	*Urgent Incontinence* Urinary tract infection	• Burning with urination • Increased frequency of urination • Pelvic pain	• Fever • Nausea • vomiting • Sepsis

### Data quality assurance

Interviewers were trained for 1 month by the project investigators before entering the field. Close supervision was conducted by the principal investigator to ensure that standard procedures were followed. A supervisor was assigned for each team to supervise and monitor the data collection process in the field, including randomization and conducting interviews in order to ensure quality of data collection in the field. The field supervisor checked all completed questionnaires daily, and mistakes were corrected immediately.

### Statistical analysis

Double data entry was cross-checked to minimize the chances of missing cases. Data were analyzed using the STATA software version 11.2. Descriptive statistics were used for determining the demographic characteristic and the prevalence of obstetric morbidities among the reproductive women. Multiple logistic regressions were used to examine the net effect of the explanatory variables on developing fistula symptoms. Here the dependent variable is coded as ‘0’ when the woman had no fistula symptoms and coded as ‘1’ if the woman complained fistula symptoms. An odds ratio greater than one indicates a positive relationship between the predictor variable and the probability of experiencing fistula symptoms, and odds ratio less than one indicates a negative relationship. The Confidence interval for all these tests was kept constant as 95% and *P* < 0.05 declared as level of significance. Data was presented in the form of tables and graphs.

## Results

An extensive field survey was conducted in different blocks of the south-eastern rural community of India. Overall, a total of 3939 eligible women were an interview, all the women gave consent to be part of the study.

### Socio-demographic characteristic and associated risk factors

In terms of socio-demographic characteristics slight higher than a quarter (27%), participants have married before the legal age, whereas generally (87%) had delivered their first child after attending a healthy reproductive age. The Majority of them were Hindu (98%), married and living with their husbands (96%). In terms of education, participant’s husbands were more educated than the participant. The study population was not economical sound as 67% of the family’s total income is less than Rs. 5000 per month. Overall among all the participants, 23.7% reported fistula like symptoms. To explore more for predictors of fistula-like symptoms a logistic regression model was adopted. The model had been adjusted all the predictors, the logistic regression model shows eleven predictors determining more chance to get fistula-like symptoms: participant’s education, husband education, family income, number of delivery, number of abortion, number of stillbirths, number of antenatal check-ups, place of delivery, walking distance from nearest health facilities, time of labor pain and more no of moves during labour. In the multiple logistic regression -adjusted model in Table 2 depicted that participants who had not undergone any formal education and primary education (AOR: 3.89; 95% CI: 2.86–5.28; *p* = 0.000) and (AOR: 1.35; 95% CI: 1.01–1.78; *p* = 0.036) respectively. Participants whose husbands not underwent any formal education (AOR: 0.36; 95% CI: 0.24–0.55; *p* = 0.000), followed by the family having monthly income less than Rs. 5000 (AOR: 0.40; 95% CI: 0.20–0.82; *p* = 0.013), participants delivered three or more times (AOR: 2.51; 95% CI: 1.86–3.39; *p* = 0.000), one or more abortion (AOR: 2.04; 95% CI: 1.12–3.71; *p* = 0.018), one or more still birth (AOR: 2.44; 95% CI: 1.30–4.57; *p* = 0.005), one or no antenatal check up (AOR: 1.76; 95% CI: 1.36–2.28; *p* = 0.000) followed by home delivery (AOR: 4.38; 95% CI: 2.31–8.30; *p* = 0.000), more than 2 h walking distance from nearest health facilities (AOR: 1.78; 95% CI: 1.32–2.40; *p* = 0.000), the women pained more than 24 h of labour (AOR: 0.15; 95% CI: 0.08–0.26; *p* = 0.000) followed by two or more unplanned moves which include home to place of delivery (AOR: 2.49; 95% CI: 1.60–3.89; *p* = 0.000) and TBA delivery at home (AOR: 0.35; 95% CI: 0.18–0.67; *p* = 0.001) are been a significant predictor determining more susceptible to get fistula-like symptoms **(**Table 2)**.**

**Table 2:** Multiple logistic regression model determining predictors for fistula symptoms (*N* = 3939)

Characteristics	No Fistula symptoms N (%)	Fistula symptoms N (%)	AOR (95%)	*P*- value
Religion				
Hindu	2934 (76.3%)	912 (23.7%)	Ref	Ref
Muslim	68 (73.1%)	25 (26.9%)	1.05 (0.63–1.73)	0.846
Education				
Graduation	685 (75.8%)	219 (24.2%)	Ref	Ref
Secondary	1135 (76.2%)	354 (23.8%)	1.23 (0.98–1.53)	0.063
Primary	949 (82.5%)	202 (17.5%)	1.35 (1.01–1.78)	0.036*
No formal education	233 (59.0%)	162 (41.0%)	3.89 (2.86–5.28)	0.000*
Husband level Education				
Graduation	165 (73%)	61 (27%)	Ref	Ref
Secondary	1110 (78.6%)	302 (21.4%)	0.77 (0.53–1.11)	0.168
Primary	1224 (74.6%)	416 (25.4%)	0.69 (0.47–1.03)	0.071
No formal education	503 (76.1%)	158 (23.9%)	0.36 (0.24–0.55)	0.000*
Family income				
< 5000	1972 (74.9%)	662 (25.1%)	0.40 (0.20–0.82)	0.013*
5000–40,000	1006 (79.5%)	259 (20.5%)	0.51 (0.25–1.03)	0.063
> 40,000	24 (60.0%)	16 (40.0%)	Ref	Ref
Age at time of marriage				
≥ 18	2215 (77.3%)	651 (22.7%)	Ref	Ref
< 18	787 (73.3%)	286 (26.7%)	0.97 (0.79–1.19)	0.817
Age at 1st Delivery				
≥ 18	2607 (76.0%)	825 (24.0%)	Ref	Ref
< 18	395 (77.9%)	112 (22.1%)	1.06 (0.78 1.45)	0.677
Number of Delivery				
One delivery	392 (83.6%)	77 (16.4%)	Ref	Ref
Two delivery	1528 (84.1%)	289 (15.9%)	0.80 (0.59–1.08)	0.149
Three delivery	1082 (65.5%)	571 (34.5%)	2.51 (1.86–3.39)	0.000*
Number of Abortion				
No abortion	2969 (76.4%)	916 (23.9%)	Ref	Ref
≥ One abortion	33 (61.1%)	21 (38.9%)	2.04 (1.12–3.71)	0.018*
Still birth				
No still birth	2978 (76.6%)	909 (23.4%)	Ref	Ref
≥ One still birth	24 (46.2%)	28 (53.8%)	2.44 (1.30–4.57)	0.005*
Number of antenatal visit				
Four	1036 (83.2%)	209 (16.8%)	Ref	Ref
Three	539 (84.6%)	98 (15.4%)	0.92 (0.69–1.22)	0.581
Two	127 (79.9%)	32 (20.1%)	1.27 (0.82–1.99)	0.276
≤ One	1300 (68.5%)	598 (31.5%)	1.76 (1.36–2.28)	0.000*
Place of Delivery				
Institute	1656 (82.8%)	344 (17.2%)	Ref	Ref
Home	1346 (69.4%)	593 (30.6%)	4.38 (2.31–8.30)	0.000*
Walking distance from nearest health facilities				
Less than half an hour	1545 (75.5%)	501 (24.5%)	Ref	Ref
Less than 1 h	1142 (77.3%)	335 (22.7%)	1.13 (0.94–1.36)	0.177
More than 2 h	315 (75.7%)	101 (24.3%)	1.78 (1.32–2.40)	0.000*
Time of labor pain				
< 12 h	2744 (75.7%)	880 (24.3%)	Ref	Ref
12–24	76 (67.9%)	36 (32.1%)	1.43 (0.89–2.28)	0.135
> 24	182 (89.7%)	21 (10.3%)	0.15 (0.08–0.26)	0.000*
Types of moves during labour				
Planned one move	1555 (83.4%)	310 (16.6%)	Ref	Ref
Emergency referred two move	42 (63.6%)	24 (36.4%)	1.52 (0.77–3.02)	0.224
Unplanned ≥Two moves	80 (62.5%)	48 (37.5%)	2.49 (1.60–3.89)	0.000*
TBA delivery	1325 (70.5%)	555 (29.5%)	0.35 (0.18–0.67)	0.001*

*significant

### Community intervention for prevention of obstetric fistula

**Fig. 1:**
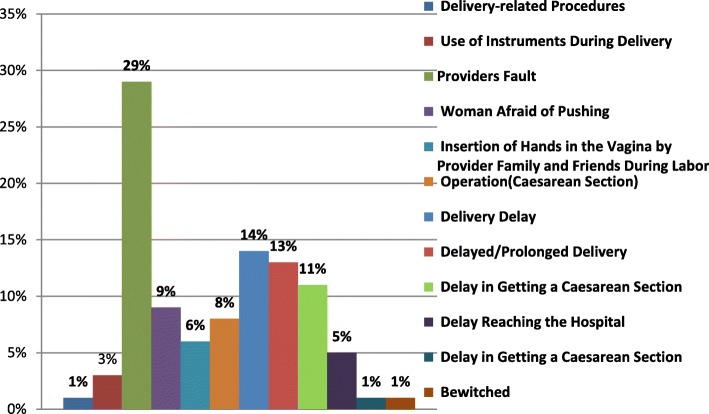
Perception of participants regarding the cause of fistula (*N* = 3939)

Knowledge and perception of the community are quite essential before planning the desired intervention for the prevention of obstetric fistula. In terms of knowledge about obstetric fistula only, 0.3% of people knew it’s a disease, a very few participants responded (0.2%) that urine incontinence symptoms after delivery may be a cause of obstetric fistula. Almost none (0.1%) of them knew that screening and surgical repair of obstetric fistula can be done free of cost in all district- level hospitals. The perception about obstetric fistula had been assessed as it depicts **(**Fig. 1**)** the majority have perceived obstetric fistula occurs because of provider’s fault followed by a delivery delay and the lowest perception was due to delivery-related procedures, delay getting caesarean section and bewitched. Along with the participant’s awareness, the community health worker’s knowledge level was also assessed through a structured training program regarding knowledge about the obstetric fistula, its identification and measured for its prevention. Simultaneously series of community training program was conducted for 3 years, results revealed that the overall percentage of post-test knowledge was more compared to the percentage of the pre-test knowledge and found statistically significant (Table 3). Hence it is observed that the training program was effective in enhancing the level of knowledge and utilizing a community-based action plan to identify the new cases, its early referral for further diagnosis and treatment. Preventive measures were taken by them to evacuate all labour cases without delay in reaching to the planned delivery place.

**Table 3:** Effectiveness of training programme on obstetric fistula (*N* = 300)

Obstetric fistula Training programme	Pre-test (x)	Post-test (y)	Effectiveness (y-x)	t-value	Level of Significance
Assessment of knowledge score of Health worker.	27.90 ± 7.72	31.54 ± 8.40	3.64 ± 0.68	−4.20	0.002*

**Fig. 2:**
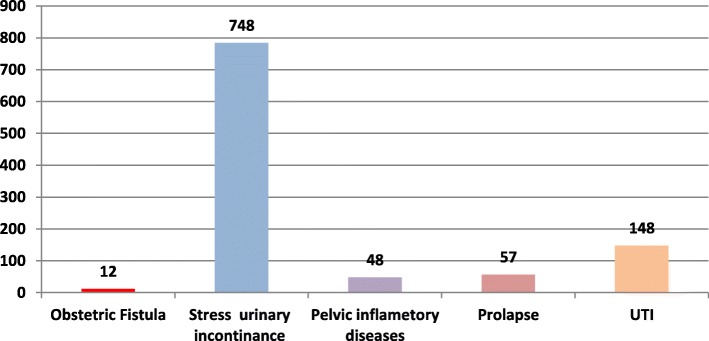
Obstetric morbidities among the reproductive women (*N* = 937**)**

Figure 2 summarises the results of the medical examination, a team of obstetrician clinically examined the participants suspected with fistula like symptoms and diagnosed 12 confirm cases of obstetric fistula, 748 cases of stress urinary inconstancies, 48 cases of pelvic inflammatory disease, 57 cases of uterine prolapsed and 148 cases of UTI. Table 4 presents details of 12 Obstetric Fistula diagnosed cases with their signs, symptoms and examination procedure.

**Table 4:** Details diagnosed cases of 12 obstetric fistula patients with their signs and symptoms and examination procedure

Sl No	Morbidities	Sign/Symptoms	Examination
O_F P1	Vesico-Vaginal Fistula	i) Continuous leaking of urine (incontinence), Vulval skin (redness, ulceration) ii) Palpation- Size,number,scarring	*Speculum examination* **i)** Fistula can be visualized
O_F P2	Vesico-Vaginal Fistula	Continuous leaking of urine (incontinence), Vulval skin (redness,ulceration) Palpation- size number, scarring.	*Speculum examination* **ii)** Fistula can be visualised
O_F P3	Recto-Vaginal Fistula	Incontinence of liquid stool/flatus	*Speculum examination* i) Fistula- exposing the posterior vaginal wall **ii)** Rectal examination
O_F P4	Vesico-Vaginal Fistula	Continuous leaking of urine (incontinence), Vulval skin (redness,ulceration) Palpation- size number, scarring	*Speculum examination* **iii)** Fistula can be visualised
O_F P5	Recto-Vaginal Fistula	Incontinence of liquid stool/flatus	*Speculum examination* iii) Fistula- exposing posterior vaginal wall **iv)** Rectal examination
O_F P6	Vesico-Vaginal Fistula	Continuous leaking of urine (incontinence), Vulval skin (redness, ulceration) Palpation- size number, scarring	*Speculum examination* **iv)** Fistula can be visualised
O_F P7	Vesico-Vaginal Fistula	Continuous leaking of urine (incontinence), Vulval skin (redness,ulceration) Palpation- size number, scarring	Speculum examination **v)** Fistula can be visualised
O_F P8	Recto-Vaginal Fistula	Incontinence of liquid stool/flatus	*Speculum examination* v) Fistula- exposing posterior vaginal wall **vi)** Rectal examination
O_F P9	Vesico-Vaginal Fistula	Continuous leaking of urine (incontinence) Vulval skin (redness,ulceration) Palpation- size number, scarring,	*Speculum examination* **vi)** Fistula can be visualised
O_F P10	Vesico-Vaginal Fistula	Continuous leaking of urine (incontinence), Vulval skin (redness,ulceration) Palpation- size number, scarring	*Speculum examination* **vii)** Fistula can be visualised
O_F P11	Vesico-Vaginal Fistula	Continuous leaking of urine (incontinence), Vulval skin (redness,ulceration) Palpation- size number, scarring	*Speculum examination* **viii)** Fistula can be visualised
O_F P12	Vesico-Vaginal Fistula	Continuous leaking of urine (incontinence), Vulval skin (redness,ulceration) Palpation- size number, scarring	*Speculum examination* **ix)** Fistula can be visualised

Health seeking practices adopted by obstetric fistula patients were analysed which shows maximum cases had not gone for routine antenatal check-up, got delayed in reaching to hospital for emergency delivery and suffered with prolonged labour, also taken three or more move from home to place of delivery. Most of them experienced delay in delivery by the care giver and reaching to the hospital due to delay in family decision **(**Table 5**).**

**Table 5:** Health seeking practices adopted by obstetric fistula patients (*N* = 12)

Characteristics	N	%
ANC Visits		
No ANC	7	58%
One ANC	5	42%
Number of moves		
No moves	4	33%
Three or move	8	67%
Family support for treatment		
No support	3	25%
Husband	9	75%
Type of delay experienced by women		
Lack of recognition of the problem by family	4	33%
Delay in seeking care	1	8%
Delay in transportation	1	8%
Delay at the hospital due to lack of equipment	2	17%
Delay by the provider at the hospital	4	33%
Post fistula condition		
Nothing changed	8	67%
Sexual &Domestic violence	2	17%
Separated /Divorced	1	8%
Husband remarried	1	8%

## Discussion

In the present study, the overall prevalence of obstetric fistula was 0.3% and the stress urinary continence was the most prevalent among the reproductive age group (15–49) of women per clinical examination.

The study findings indicated that though the prevalence of fistulasymptom among reproductive women has reduced (0.3% prevalence), it continues to be a problem for women living in rural areas, and it is preventable [16]. This is supported by other studies that have found a low prevalence of lifetime fistula symptoms (0.3–1.8% across India) among the reproductive age group women and higher incidence in rural residents [16–21]. Our study determined the prevalence of gynaecological problems among 3939 women of the reproductive age group shows a high prevalence of urinary problems like stress urinary incontinence; (19.90%), Pelvic inflammatory diseases; (1.21%), Prolapse; (1.44%) and Urinary tract infection; (3.75%). Finding of our study are similar to a community-based cross-sectional study conducted in an urban area of Belgaum, Karnataka, India among 400 married women of the reproductive age group, which reveals a high prevalence of reproductive tract infections (70%) among which 24.25%had chronic PID [12].

In low income countries, several social, cultural and health system factors contribute to the increasing risk of obstetric fistula such as lack of emergency obstetric care, child marriage associated with early pregnancy, poverty, malnutrition and poor health services [18, 19]. Commonly reported primary risk factors for obstetrical fistula include the place of birth and presence of a skilled birth attendant, the duration of labor and early marriage, [22–24]. Also women with no antenatal care and those who delivered at home found to be in more risk of developing obstetric fistula [21, 25].

In this study, the occurrence of fistulae like symptoms was associated with some demographic variables such as education, husband level education and high parity, similar to findings from other studies [22, 26]. Lack of antenatal care, prolonged labor and poor health seeking behaviour such as delay in accessing emergency care due to taking more than one move from home to reach the delivery place, no antenatal visit, lack of decision making in emergency by family members were associated with the occurrence and prevalence of obstetric fistula. Previous studies also reported delay in seeking or receiving care for more than 6 h of labor onset, taking more than 2 h to reach a health facility and long duration of labor (> 24 h) are associated risk factors developing obstetric fistula [23].

A study reported women with fistula symptoms also reported the experience of sexual and physical violence much more often than women without such symptoms [1], which is also consistent with the present findings, this is worrisome because a lack of awareness affects their ability to availing the proper treatment. As we are growing with advanced technology and well equipped with resources for providing quality maternal care, hence this is the high time it should be eradicated from all parts of the county for which community sensitization by community stakeholders is essential. Study suggested that regular sensitization training programme and follow up sessions are significant components for experiencing favourable pregnancy outcome and will reduce maternal and neonatal complication in rural community level [24].

Women’s autonomy is another determinant of obstetric fistula [27, 28]. In Indian society, the male is habitually the ones to decide important family matters, whereas women do not take any decision even related to their own health. Delay in reaching to the obstetric care facility for safe and timely intervention for women presenting with obstructed labour was reported in this study, which was mostly due to lack of autonomy in decision making during emergency by the pregnant women.

A lack of awareness among women participants about obstetric fistula, cause of occurrence and availability of free treatment option were evident in the current study, which is also reported in a study conducted in china [29]. A strategic combination of both health-system based such as improved access to emergency care, providing safe and timely intervention for preventing obstructed or prolonged labour and population based strategies such educating local community about culture, social and physiological factors that increases risk of OF may be more effective in eradicating fistula in developing countries where women are unable to access the health care system [30]. To the best of our knowledge, this is the first population cum health- system based strategic study in eastern rural India that comparatively investigated the potential factors such as demographic and reproductive health characteristics of women were associated with obstetric fistula. Fistula training was extended to the skilled birth attendants and other community health workers for taking need based action for prevention of prolonged labour, simultaneously, OF prevalent local community was educated about cultural, social and psychological factors that influences and contribute to fistula. Similar evidence is supported in other relevant literatures [24, 27–30].

It is possible that community-based studies represent an underestimate of the prevalence of obstetric fistula, as fistulae are generally more commonly found in regions where there is no access to obstetric care, and may be difficult to reach [1]. One of the findings of present study indicated that there is lack of reporting of the obstetric fistula due to the poor medical facility at community level, less awareness and understanding among women and lack of knowledge among community health workers on early identification and referral of women for diagnosis and treatment. This study was conducted with an aim to explore the various cultural, social and health related factors that associated with OF and extend periodic fistula prevention training to sensitise the health workers and pregnant women for better birth preparedness and accessing the emergency obstetric care so that obstetric fistula cases can be eradicated in the community level in developing counties like India.

Raising awareness regarding prevention of obstructed labour and the benefit of early transport to a health care facility in cases of prolonged labour is one of the important interventions and protective measures for reducing the labour-related morbidities like developing a fistula [31]. The current study identified that adequate birth preparedness and health-seeking behaviours were associated with less chance of developing fistulae like symptoms and other chronic morbidities due to obstructed labour.

Ultimately, it is anticipated that our novel research findings will be utilized by reproductive health care providers, health care policy makers, and researchers in order to implement the obstetric fistula eradication action plan in the community level and plan for referral system for obstetric fistula repair and social reintegration of women after treatment.

### Study limitations

Firstly, the limitation of this study is that only one rural block was selected in the south-eastern rural community of India, which may limit the validity of study outcome. Secondly, confirmatory diagnosis of fistula cases was not done by dye test, only speculum examination was done after pre-screening of cases in community level., Thirdly, study addressed effectiveness of training programme here in terms of measuring knowledge level of community health workers only and detail implementation of obstetric fistula action plan and its outcome will be presented separately.

## Conclusion

Our present study showed 0.3% prevalence of obstetric fistula and more chance of occurrence found to be associated with inadequate antenatal care services, home delivery, poorly equipped resources for emergency obstetric care in health centres and more moves to the delivery place. In addition the obstetric fistula symptoms were more associated with their demographic factors. This study also evaluated the poor level of awareness among rural women and field health workers regarding obstetric fistula. Fistula patients are living indicators of maternal health care, and a solution to the problem will ultimate the provision of essential obstetric care services. Finally, our study recommends that a community-level action plan must be implemented for the provision of essential and emergency obstetric care services for pregnant women at all health care levels for the prevention of obstructed and prolonged labour, which is the most common cause of obstetric fistula. Addressing socio-demographic determinants will certainly contribute towards reducing the occurrence of obstetric fistula. Also, community health workers must be trained enough for early identification and referral of obstetric fistula cases for immediate treatment.

## Supplementary information

**Additional file 1.** Obstetric fistula pre-screening interview schedule.

## Data Availability

The data sets generated and/or analysed during the current study are not publicly available because of maintaining confidentiality of the women diagnosed with obstetric fistula which is still considered a stigma in the public health, but are readily available from the corresponding author on reasonable request. The authors agreed to share the raw data on request recognizing the benefits of such transparency.

## Abbreviations

ANC: Antenatal care
OF: Obstetric fistula
OR: Odds ratio
SPSS: Statistical package for social science
UNFPA: United nation population fund
