# Supplementary material for: Prevalence and risk factors of obstetric fistula: implementation of a need-based preventive action plan in a South-eastern rural community of India
Source: BMC Womens Health. 2020 Mar 4;20:40. doi: 10.1186/s12905-020-00906-w (PMC7055058; doi:10.1186/s12905-020-00906-w)
Supplement: Supplementary file 1 — Additional file 1. Obstetric fistula pre-screening interview schedule. [file 12905_2020_906_MOESM1_ESM.pdf]

PRE-SCREENING INTERVIEW- OBSTETRIC FISTULA

SCREENING ID NUMBER					

**Name**(ନାମ):	**Husband Name**(ସ୍ୱାମୀଙ୍କ ନାମ):

**1. What is Your Age**(ଆପଣଙ୍କ ବୟସ)**?**	

**2. Religions** (ଧର୍ମ)**:** (If Other, Specify)(ଯଦି ଅନ୍ୟ, ନିର୍ଦ୍ଦିଷ୍ଟ କରନ୍ତୁ,_____)	Hindu(ହିନ୍ଦୁ)	0	Christian(ଖ୍ରୀଷ୍ଟିୟାନ୍)	...1
	Muslim(ମୁସଲମାନ)	2	Other(ଅନ୍ୟ)	...3

**3. Marital Status:** (ବୈବାହିକ ଅବସ୍ଥା)	Married(ବିବାହିତ)	...0	Divorced(ଛାଡ଼ପତ୍ର ପ୍ରାପ୍ତ)	...1
	Widowed(ବିଧବା)	...2	Single(ଅବିବାହିତ)	...3

**4. Education** (ଶିକ୍ଷା)**:**(If Other, Specify)(ଯଦି ଅନ୍ୟ, ନିର୍ଦ୍ଦିଷ୍ଟ କରନ୍ତୁ,_____)	None(କିଛି ନୁହେଁ)	0	Primary(ପ୍ରାଥମିକ)	...1
	Secondary(ମାଧ୍ୟମିକ)	2	Higher Secondary (ଉଚ୍ଚ ମାଧ୍ୟମିକ)	...3
	Graduation(ସ୍ନାତକ)	4	Don't Know(ଅଜ୍ଞାତ)	...9

**5. Husband Level of Education:** (ସ୍ୱାମୀଙ୍କ ଶିକ୍ଷା) If Other, Specify)(ଯଦି ଅନ୍ୟ, ନିର୍ଦ୍ଦିଷ୍ଟ କରନ୍ତୁ,_____)	None(କିଛି ନୁହେଁ)	0	Primary(ପ୍ରାଥମିକ)	...1
	Secondary(ମାଧ୍ୟମିକ)	2	Higher Secondary (ଉଚ୍ଚ ମାଧ୍ୟମିକ)	...3
	Graduation(ସ୍ନାତକ)	4	Don't Know(ଅଜ୍ଞାତ)	...9

**6. Family Income** (ପାରିବାରିକ ଆୟ)**:**		Income $\leq$ Rs.5000 (≤ ଟ ପାଞ୍ଚହଜାର)	...0
Income Rs.5,000–10,000 (ଟ ପାଞ୍ଚହଜାରରୁ ଦଶହଜାର)	1	Income Rs.20,000–40,000 (ଟ କୋଡ଼ିଏ ହଜାରରୁ ଚାଳିଶି ହଜାର)	3
Income Rs.10,000–20,000 (ଟ ଦଶ ହଜାରରୁ କୋଡ଼ିଏ ହଜାର)	2	Income > Rs.40,000 (> ଟ ଚାଳିଶି ହଜାର)	4

REPRODUCTIVE HEALTH (ପ୍ରଜନନ ସ୍ୱାସ୍ଥ୍ୟ)

7. Respondents' Age at the Time of Marriage (ଜବାବଦାତାଙ୍କ ବିବାହ ବୟସ)**:**

Less than 18 years old (18 ବର୍ଷରୁ କମ୍)		21-30years old (21 ରୁ 30 ବର୍ଷ ମଧ୍ୟରେ)	
18-20years old (18 ରୁ 20 ବର୍ଷ ମଧ୍ୟରେ)		31-40years old (31 ରୁ 40 ବର୍ଷ ମଧ୍ୟରେ)	
Respondents Age at the Time of 1st Delivery(ଜବାବ ଦାତାଙ୍କ ପ୍ରଥମ ପ୍ରସୂତିର ସମୟ)			

**8. Have You Ever Given Birth Before** (ଆପଣ ଛୁଆ ଜନ୍ମ କରିଛନ୍ତି କି)**?** If No, **Skip to #12**	No (ନା)	0	Yes (ହଁ)	1

9. Number of Deliveries of Respondents:

One Delivery(ଗୋଟିଏ ପ୍ରସୂତି)	0	Two Deliveries(ଦୁଇଟି ପ୍ରସୂତି)	1
Three (3) or More Deliveries (ତିନି(3) କିମ୍ବା ଅଧିକ ପ୍ରସୂତି)			2

10. How Many Sons or Daughters to Whom You have Given Birth are Alive' (ଆପଣଙ୍କର କେତେ ଜଣ ପୁଅ/ଝିଅ ବଞ୍ଚିଛନ୍ତି)**?**

Sons(ପୁଅ)	1	Daughters(ଝିଅ)	2

11. Have You Ever Given Birth to a Boy or Girl Who Was Born Alive but Later Died(ଆପଣଙ୍କର କେହି ପୁଅ/ଝିଅ ଜନ୍ମପରେ ମରିଯାଇଛନ୍ତି କି)**?**

No(ନାଁ)	0	Yes(ହଁ)	1

12. How Many Boys or Girls Have Died Like This(ଏପରି କେତେ ଜଣ ପୁଅ/ଝିଅ ମରିଛନ୍ତି)?

Boys Dead(ପୁଅ ମରିଛନ୍ତି)	1	Girls Dead(ଝିଅ ମରିଛନ୍ତି)	2

13. Have You Ever Lost a Pregnancy? It Might be Spontaneously or When You or Someone Else Had to do Something To End the Pregnancy (ଆପଣ କେବେ ଗର୍ଭାବସ୍ଥା ନଷ୍ଟ ହେଇଛି କି? ଆପଣ ସ୍ୱେଚ୍ଛାପ୍ରବୃତ୍ତ ବା କାହା କଥାରେ ପଡ଼ି ଗର୍ଭ ନଷ୍ଟ କରିଛନ୍ତି କି)**?**

If No, **Skip #14**	No(ନାଁ)	0	Yes(ହଁ)	1

**14. How Many Pregnancies Have You Lost in Your Lifetime** (ଆପଣ ଜୀବନକାଳରେ କେତୋଟି ଗର୍ଭ ନଷ୍ଟ କରିଛନ୍ତି)**?**	

15. Have You Ever had a Stillbirth(ଆପଣଙ୍କର କେବେ ମୃତଛୁଆ ଜନ୍ମ ହୋଇଛନ୍ତି କି)**?**

No(ନାଁ)	0	Yes(ହଁ)	1

**How many Stillbirths have You had in Your Lifetime** (ଆପଣ ଏପରି କେତୋଟି ମୃତଛୁଆ ଜନ୍ମ କରିଛନ୍ତି)**?**	

FISTULA LIKE SYMPTOMS(ଭଗନ୍ଦର ଜନିତ ଲକ୍ଷଣ)

16. Have You Ever Experienced a Constant Leakage of Urine or Stool from Your Vagina During The Day and Night(ଆପଣ କେବେ ଦିନ ଏବଂ ରାତିରେ ଯୋନିରୁ ଏକସମୟରେ ପରିସ୍ରା କିମ୍ବା ଝାଡ଼ା ବହିବାର ଅନୁଭବ କରିଛନ୍ତି କି)**?**

If No, **Skip to #33**	No(ନାଁ)	0	Yes(ହଁ)	1

17. Did this Problem Occur Within the Last 12 Months(ଏହି ଅସୁବିଧା ବିଗତ ବାରମାସ(12) ହେଲା ଚାଲୁଅଛି କି)**?**

If Yes, **Skip to #20**	No(ନାଁ)	0	Yes(ହଁ)	1

**18. What Year Did This Problem Occur**(କେଉଁ ବର୍ଷ ଏହି ଅସୁବିଧା ଆରମ୍ଭ ହୋଇଥିଲା)**?**	

19. a) Did This Problem Occur After a Delivery(ଏହି ଅସୁବିଧା ପ୍ରସବ ପରେ ଆରମ୍ଭ ହୋଇଛି କି)**?**

If Yes, **Skip to #20**	No(ନାଁ)	0	Yes(ହଁ)	1

b) Did This Problem Occur after an Operation in your Pelvic Area(ଏହି ଅସୁବିଧା ଆପଣଙ୍କର ତଳି ପେଟର ଅସ୍ତ୍ରପଚାର ପରେ ହେଇଛି କି) **? (Pelvic Surgery)**

If Yes, **Skip to #28**	No(ନାଁ)	0	Yes(ହଁ)	1

**c) Did this Problem Occur After Some Other Event**(ଏହି ଅସୁବିଧା କିଛି ଗୁରୁତ୍ୱପୂର୍ଣ୍ଣ ଘଟଣା ପରେ ହୋଇଛି କି)**?**	No(ନାଁ)	0	Yes(ହଁ)	1

If **OTHER**, Please Specify(ଯଦି ଅନ୍ୟ, ନିର୍ଦ୍ଦିଷ୍ଟ କରନ୍ତୁ) 2

CONFIRMATION QUESTION FOR THE TOOL(ନିଶ୍ଚିତୀକରଣ ପ୍ରଶ୍ନ)

Did You Have Problem After Delivery(ଆପଣଙ୍କର ପ୍ରସବପରେ ଅସୁବିଧା ହୋଇଛି କି)**?**

If No, **Skip to #29**	No(ନାଁ)	0	Yes(ହଁ)	1

**20. How Many ANC Check-ups You have Attended** (ଆପଣ କେତେଥର ଗର୍ଭଧାରଣ ସମ୍ବନ୍ଧୀୟ ଚିକିତ୍ସା କରାଇଛନ୍ତି)**:**	No(ନାଁ)........0
ONE(ଏକ)................1	TWO(ଦୁଇ)................................2
THREE(ତିନି).............3	FOUR(ଚାରି)...............................4

21. Did This Problem Occur After a Normal Labor and Delivery, or After a Very Difficult Labor and Delivery(ଏହି ଅସୁବିଧା ପ୍ରସୂତି ପରେ ବା କଷ୍ଟଜନିତ ପ୍ରସୂତି ପରେ ବା ଗର୍ଭପରେ ହୋଇଥିଲା କି)**?**

Normal Labor/ Delivery (ସାମାନ୍ୟ କଷ୍ଟ/ପ୍ରସବ)	1	Very Difficult Delivery (ଅତ୍ୟନ୍ତ କଷ୍ଟଜନିତ ପ୍ରସବ)	2

22. Where did the Delivery Take Place(ଆପଣଙ୍କର ପ୍ରସବ କେଉଁଠାରେ ହୋଇଥିଲା)**?**

Home (ଘରେ)	0	Institutional Delivery(ଚିକିତ୍ସାଳୟ)	1

23. Walking Distance from Nearest Health Facility(ଆପଣଙ୍କ ଘରଠାରୁ ଗ୍ରାମ୍ୟ ଚିକିତ୍ସାକେନ୍ଦ୍ର କେତେ ଦୂର ଅଟେ)**:**

Less Than Half Hour (ଅଧଘଣ୍ଟାରୁ କମ୍)	0	Less Than Two Hour (ଦୁଇଘଣ୍ଟାରୁ କମ୍)	2
Less Than One Hour (ଏକଘଣ୍ଟାରୁ କମ୍)	1	More Than Two Hour (ଦୁଇଘଣ୍ଟାରୁ ଅଧିକ)	3

24. How Long After the Labor Pains Began did You Go to the Facility(ପ୍ରସୂତି ଯନ୍ତ୍ରଣା ଆରମ୍ଭହେବାର କେତେସମୟ ପରେ ଆପଣ ଚିକିତ୍ସାକେନ୍ଦ୍ରରେ ପହଁଞ୍ଚିଥିଲେ)?

<12 Hours(ବାର ଘଣ୍ଟାରୁ କମ୍)	0	>24 Hours(ଚବିଶ ଘଣ୍ଟାରୁ ଅଧିକ)	2
12-24 Hours (ବାରରୁ ଚବିଶ ଘଣ୍ଟାର ମଧ୍ୟରେ)	1	I don't Know(ମୁଁ ଜାଣି ନାହିଁ)	9

25. Did You Get a Caesarean Section at the Facility(ସେହି ଚିକିତ୍ସାକେନ୍ଦ୍ରରେ ସିଜରିଆନର ସୁବିଧା ଉପଲବ୍ଧ ଥିଲା କି)**?**

No(ନାଁ)	0	Yes(ହଁ)	1

**26. Was this Baby Born Alive**(ଆପଣଙ୍କର ନବଜାତ ଶିଶୁ ଜୀବୀତ ଅବସ୍ଥାରେ ଜନ୍ମ ହୋଇଥିଲା କି)**?**	No(ନାଁ)	0	Yes(ହଁ)	1

**27. After Which Delivery Did This Occur**(କେଉଁ ପ୍ରସବ ପରେ ଏହି ଅସୁବିଧା ଆରମ୍ଭ ହେଲା)**?**	Delivery Number(ପ୍ରସବ ସଂଖ୍ୟା)	

**28. How Many Days After Did the Leakage Start** (କେତେ ଦିନରୁ ଏମିତି କ୍ଷରଣ ହେଉଛି)**?** (Answer to Question #19) **Number of Days After the Precipitating Event** (Enter 99, If More than 99 Days)	

29. Have You Sought Treatment for This Condition (ଏହି ଅସୁବିଧା ପାଇଁ ଆପଣ କିଛି ଚିକିତ୍ସା ନେଇଛନ୍ତି କି)**?**

If No, **Skip to #31**	No(ନାଁ)	0	Yes(ହଁ)	1

30. From Whom Did You Last Seek Treatment(ଆପଣ କାହାଠାରୁ ଚିକିତ୍ସୀତ ହୋଇଥିଲେ)**?**

Health Professional Doctor/ Clinical Officer(ଡ଼ାକ୍ତର)	0
Nurse/ Midwife(ଧାଇ)	1
Patient Attendant(ରୋଗୀ ପରିଚାରକ)	2
Other Person-Untrained Village Doctor(ତାଲିମ ପାଇନଥିବା ଗ୍ରାମ୍ୟ ଚିକିତ୍ସକ)	3
Pharmacists(ଭେଷଜଜ୍ଞ)	4
If Other, Please Specify(ଯଦି ଅନ୍ୟ, ନିର୍ଦ୍ଦିଷ୍ଟ କରନ୍ତୁ)	5

31. Why Have You Not Sought Treatment? (ଆପଣ କେଉଁ କାରଣରୁ ଚିକିତ୍ସୀତ ହୋଇନାହାଁନ୍ତି)

Did not Know how it could be fixed (କିପରି ଭଲହେବ ମୁଁ ଜାଣିନାହିଁ)			0
Do not Know Where to Go(କେଉଁଠିକି ଜିବି ମୁଁ ଜାଣିନାହିଁ)			
Too Expensive(ବ୍ୟୟବହୁଳ)	1	Too Far(ବହୁତଦୂର)	2
Poor Quality of Care(ନିମ୍ନଧରଣର ସେବା)			3
Could not Get Permission(ଅନୁମତି ମିଳୁନାହିଁ)			4
Embarrassment(ଲଜ୍ଜିତକରଣ)			5
If Other, Please Specify(ଯଦି ଅନ୍ୟ, ନିର୍ଦ୍ଦିଷ୍ଟ କରନ୍ତୁ)			6

32. Did the Treatment Stop the Problem(ଏହି ଚିକିତ୍ସା ଆପଣଙ୍କ ଅସୁବିଧାକୁ ଦୂରିଭୁତ କରିଛି କି)**?**

Yes, No More Leakage At All (ହଁ, କ୍ଷରଣ ଆଉ ହେଉ ନାହିଁ)	0	No, Still Have Problem (ନା, ଏବେବି ଅସୁବିଧା ହେଉଅଛି)	...2
Yes, But Still Some Leakage (ହଁ, କିଛି ମାତ୍ରାରେ କ୍ଷରଣ ହେଉଛି)	1		

33. Are There Any (Other) Women in Your Household Who Suffer from Vesicovaginal Fistula/ Obstetric Fistula(ପରିବାରର ଅନ୍ୟକୌଣସି ମହିଳା ଭେସିକୋଭେଜାଇନାଲ/ଓବଷ୍ଟେଟ୍ରିକ ଫିଷ୍ଟୁଲା ଦ୍ଵାରା ପୀଡ଼ିତ କି)**?**

No(ନାଁ)	0	Yes(ହଁ)	1

34. How Many (Other) Women in your Household Suffer from Vesicovaginal Fistula/ Obstetric Fistula(ପରିବାରର କେତେଜଣ ମହିଳା ଭେସିକୋଭେଜାଇନାଲ/ଓବଷ୍ଟେଟ୍ରିକ ଫିଷ୍ଟୁଲା ଦ୍ଵାରା ପୀଡ଼ିତ ଅଛନ୍ତି)**?**If No, **Skip to #35**

Number(ସଂଖ୍ୟା)		Don't Know(ଅଜ୍ଞାତ)	9	No(ନାଁ)	0

35. Did She/ They Undergone Any Screening or Treatment or Are They Planning to Do(ସେ/ସେମାନେ କୌଣସି ପ୍ରକାର ଚିକିତ୍ସା ନେଇଛନ୍ତି କିମ୍ବା ଚିକିତ୍ସୀତ ହେବାପାଇଁ ଯୋଜନା କରୁଛନ୍ତି କି)**?**

Don't Know(ଅଜ୍ଞାତ)	9	No(ନାଁ)	0	Yes(ହଁ)	1

COMMUNICATION(ଯୋଗାଯୋଗ)

36. Communication Devices Available at your Home(ଘରେ ଉପଲବ୍ଧ ଯୋଗାଯୋଗ ଉପକରଣ)**.**

		Radio(ବେତାର ଯନ୍ତ୍ର)	1
Television(ଦୂରଦର୍ଶନ)	...2	Mobile(ଦୂରଭାଷା ଯନ୍ତ୍ର)	3

KNOWLEDGE ASSESSMENT & PERCEPTION OF CAUSE O.F.

37. The Continuous Leakage of Urine or Feces or Both Through Woman's Private Part is Called Fistula(ସ୍ତ୍ରୀଲୋକଙ୍କର ଯୌନାଙ୍ଗରୁ ଅବିରତ ପରିସ୍ରା/ମଳ କିମ୍ବା ଉଭୟ ନିଃସରଣ ହେବା ଭଗନ୍ଦର ଅଟେ)**.**

Don't Know(ଅଜ୍ଞାତ)	9	No(ନାଁ)	0	Yes(ହଁ)	1

38. This Condition Can be Completely Treated Through Surgical Operation in the Hospital(ଏହି ଅସୁବିଧାର ଚିକିତ୍ସା ଉପଚାର ଶଲ୍ୟ ଅସ୍ତ୍ରୋପଚାର ମାଧ୍ୟମରେ ଚିକିତ୍ସାଳୟରେ ସଂମ୍ପୂର୍ଣ୍ଣ ଭାବରେ ହୋଇପାରିବ)**.**

Don't Know(ଅଜ୍ଞାତ)	9	No(ନାଁ)	0	Yes(ହଁ)	1

39. Woman with Other Forms of Leakage Not Related to Fistula Will be Referred to Other Hospitals to Get Treatment, Where They Shall Bear the Cost of Transportation and Operation(ଯେଉଁ ସ୍ତ୍ରୀଲୋକଙ୍କର କ୍ଷରଣ ଅସୁବିଧା ଭଗନ୍ଦର ସଂମ୍ପର୍କିତ ନୁହେଁ ସେମାନଙ୍କୁ ଅନ୍ୟ ଚିକିତ୍ସାଳୟକୁ ପ୍ରେରଣ କରାଯିବ, ଯେଉଁଠାରେ ସେମାନଙ୍କୁ ପରିବହନ ଓ ଅସ୍ତ୍ରୋପଚାରର ଖର୍ଚ୍ଚ ବହନ କରିବାକୁ ପଡ଼ିବ)**.**

Don't Know(ଅଜ୍ଞାତ)	9	No(ନାଁ)	0	Yes(ହଁ)	1

40. The Women Who Are Identified with This Condition at the Screening Will be Operated Free of Charge(ଚିହ୍ନଟପ୍ରକ୍ରିୟା ଦ୍ଵାରା ଚିହ୍ନିତ ହୋଇଥିବା ସ୍ତ୍ରୀଲୋକମାନଙ୍କୁ ବିନାମୂଲ୍ୟରେ ଚିକିତ୍ସିତ କରାଯିବ)**.**

Don't Know(ଅଜ୍ଞାତ)	9	No(ନାଁ)	0	Yes(ହଁ)	1

41. Screening Should Be Done to Identify Fistula(ଭଗନ୍ଦରର ଚିହ୍ନଟୀକରଣ ପାଇଁ ଚିହ୍ନଟପ୍ରକ୍ରିୟା ଧାର୍ଯ୍ୟ ଅଟେ)**.**

Don't Know(ଅଜ୍ଞାତ)	9	No(ନାଁ)	0	Yes(ହଁ)	1

42. Who Will Accompany You For Treatment(ଚିକିତ୍ସା ପାଇଁ ଆପଣଙ୍କ ସାଙ୍ଗରେ କିଏ ଯିବେ)**?**

Alone/ Self(ଏକାକୀ)	0	Husband(ସ୍ଵାମୀ)	1
Mother/ Father(ମା/ବାପା)	2	Sister/ Brother(ଭଉଣୀ/ଭାଇ)	3
Friend/Acquaintance (ବନ୍ଧୁ/ଚିହ୍ନାଜଣା)	4	If Other, Please Specify (ଯଦି ଅନ୍ୟ, ନିର୍ଦ୍ଦିଷ୍ଟ କରନ୍ତୁ)	5

43. What Are Your Perceptions of Causes of Fistula(ଭଗନ୍ଦରର କାରଣ ବିଷୟରେ ଆପଣଙ୍କ ମନ୍ତବ୍ୟ କଣ)**?**

Delivery-related Procedures (ପ୍ରସବ ସଂମ୍ବନ୍ଧିତ ପ୍ରକ୍ରିୟା)	...0
Use of Instruments During Delivery(ପ୍ରସବ ସମୟରେ ବ୍ୟବହୃତ ଉପକରଣ)	...1
Providers Fault(ସେବାଦାତାଙ୍କ ତ୍ରୁଟି)	...2
Woman Afraid of Pushing(ଛୁଆ ଠେଲିବାରେ ପ୍ରସୂତିଙ୍କ ଭୟ)	...3
Insertion of Hands in the Vagina by Provider Family and Friends During Labor(ପ୍ରସବ ସମୟରେ ପାରିବାରିକ ଏବଂ ବନ୍ଧୁ ଲୋକଙ୍କର ଯୋନିରେ ହାତ ଅନ୍ତର୍ନିବେଶନ)	...4
Operation (Caesarean Section) ଅସ୍ତ୍ରୋପଚାର(ସିଜରିଆନ୍ ବିଭାଗ)	...5
Delivery Delay (ପ୍ରସବରେ ବିଳମ୍ବ)	...6
Delayed/ Prolonged Delivery(ବିଳମ୍ବିତ/ଦୀର୍ଘକାଳୀନ ପ୍ରସବ)	...7
Delay in Getting a Caesarean Section (ସିଜରିଆନ୍ ବିଭାଗକୁ ଯିବାରେ ବିଳମ୍ବ)	...8
- Delay Reaching the Hospital(ଚିକିତ୍ସାଳୟ ପହଁଞ୍ଚିବାରେ ବିଳମ୍ବ)	...9
- Bewitched(ଆଶ୍ଚର୍ଯ୍ୟଜନକ/ମନ୍ତ୍ରମୁଗ୍ଧ)	..10

44. Family Members Perceptions of Causes of Fistula(ଭଗନ୍ଦରର କାରଣ ବିଷୟରେ ପରିବାର ଲୋକଙ୍କର ମନ୍ତବ୍ୟ)**.**

Bewitchment (ଯାଦୁ)	0
Use of Instruments during Delivery/ Provider Mistake(ପ୍ରସବ ସମୟରେ ବ୍ୟବହୃତ ଉପକରଣ/ସେବାଦାତାଙ୍କ ତ୍ରୁଟି)	1
Delivery Delay (ପ୍ରସବରେ ବିଳମ୍ବ)	2
God's Will (ଭଗବାନଙ୍କ ଇଛା)	3
Failure to Deliver at a Hospital/ Being Too Young/ Lack of Care by Provider (ଚିକିତ୍ସାଳୟରେ ପ୍ରସବ କରିବାରେ ଅକ୍ଷମ/ଅତ୍ୟନ୍ତ ଯୁବା ଅବସ୍ଥା/ସେବାଦାତାଙ୍କ ଯତ୍ନରେ ଅବହେଳା)	4

HEALTH SEEKING PRACTICES(ସ୍ଵାସ୍ଥ୍ୟପରୀକ୍ଷା ଅଭ୍ୟାସ)

**1. Have You Gone For Antenatal Visits**(ଆପଣ ପ୍ରସବର ପୂର୍ବକାଳୀନ ପରୀକ୍ଷା କରାଇଛନ୍ତି କି)**?**	No (ନାଁ)	...0	Yes (ହଁ)	1
If Yes Specify No of Visits and Weeks of Pregnancy(ଯଦି ହଁ,				

2. Number of Moves Made by You from Initiation of Labour to Final Delivery(ପ୍ରାରମ୍ଭିକ ପ୍ରସବ ଶ୍ରମଠାରୁ ପ୍ରସବ ମଧ୍ୟରେ ଆପଣ କେତେଥର ସ୍ଵାସ୍ଥକେନ୍ଦ୍ର ଯାଇଛନ୍ତି)**?**

No Move(ଆଡ଼େ଼ାଂ ଯାଇନାହିଁ)	.0	1 Move(ଏକ ଥର)	.1	2 Move(ଦୁଇ ଥର)	.2
3 Move(ତିନି ଥର)	3	>3 Move(ତିନିଥରରୁ ଅଧିକା)			4

3. Types of Delay Experienced by Women with Fistula(ଭଗନ୍ଦରଦ୍ଵାର ଆକ୍ରାନ୍ତ ମହିଳାଙ୍କ ଅନୁଭୂତ ବିଳମ୍ବର ପ୍ରକାର)

Lack of Recognition of Problem by Woman or Friend/ Family(ସମସ୍ୟା ଚିହ୍ନଟୀକରଣରେ ସ୍ତ୍ରୀ କିମ୍ବା ବନ୍ଧୁ/ପରିବାର ଲୋକଙ୍କର ବିଳମ୍ବନା)	..0
Delay by TBAs in Identifying Problem and or Taking Action(ସମସ୍ୟା ଚିହ୍ନଟୀକରଣ ଏବଂ କାର୍ଯ୍ୟକରଣ ରେ ପାରଂମ୍ପରିକ ଜନ୍ମସେବକର ବିଳମ୍ବନା)	..1
Delay in Seeking Care(ଯତ୍ନ ନେବାରେ ବିଳମ୍ବନା)	..2
Delay in Transportation(ନେବାଆଣିବାରେ ବିଳମ୍ବନା)	..3
Delay at Site Due to Lack of Supplies, Equipment, etc.(ଉପକରଣ ଓ ଯନ୍ତ୍ରପାତିର ଅଭାବଜନିତ ବିଳମ୍ବନା)	..4
Delay by Provider at the Site of Hospital(ଚିକିତ୍ସାଳୟରେ ସେବାଦାତାଙ୍କ ବିଳମ୍ବନା)	..5

**4. Family Support for Treatment** (ଚିକିତ୍ସାଲାଗି ପାରିବାରିକ ସହଯୋଗ)				Self (ସ୍ଵୟଂ)	1
Husband/Children (ସ୍ଵାମୀ/ପିଲାଛୁଆ)	.2	Relatives (ପରିବାର ବର୍ଗ)	..3	Friends/ Neighbour (ବନ୍ଧୁ/ପଡ଼ୋଶୀ)	4

5. Have You Taken Any Other Type of Treatment for Fistula(ଭଗନ୍ଦର ପାଇଁ ଆପଣ ଅନ୍ୟକୌଣସି ଚିକିତ୍ସା କରାଇଛନ୍ତି କି)**?**

If Yes, Please Specify ଯଦି ହଁ, ନିର୍ଦ୍ଦିଷ୍ଟ କରନ୍ତୁ................2	No(ନାଁ)	0	Yes(ହଁ)	1

6. What is the Impact of Post-Fistula Condition in Your Life(ପାରିବାରିକ ଜୀବନରେ ଭଗନ୍ଦର ପରବର୍ତ୍ତୀ ପ୍ରଭାବ)**?**

Nothing Change(କିଛି ବଦଳି ନାହିଁ)	...0	Husband Remarried (ସ୍ଵାମୀଙ୍କର ପୁନର୍ବିବାହ)	2
Domestic Violence(ଘରୋଇ ହିଂସା)	...1	Divorced(ଛାଡ଼ପତ୍ର)	3
Do Not Wish to Respond(କହିବାକୁ ଇଛାପ୍ରକାଶ କରୁନାହିଁ)			4
